# Microstructural Control, Property Trade-Offs and Emerging Design Strategies in Conventional Aluminum Alloys

**DOI:** 10.3390/ma19183901

**Published:** 2026-09-14

**Authors:** Shuai Zhang, Bingbing Li, Zhaofeng Wang, Jiawei Han, Qiang Shi

**Affiliations:** 1 School of Mechanical Engineering, Liaoning Institute of Science and Engineering, Jinzhou 121010, China; 2 School of Materials Science and Engineering, Dalian Jiaotong University, Dalian 116028, China; 3 Faculty of Electrical and Control Engineering, Liaoning Technical University, Huludao 125105, China

**Keywords:** traditional aluminum alloy, strengthening mechanism, precise microstructure control, recycled raw materials, data-driven method

## Abstract

Traditional aluminum alloys have a mature industrial system, but their properties are still constrained by factors such as precipitation behavior, the grain boundary state, solidification defects, and residual impurities. This paper systematically reviews the microstructure formation, strengthening mechanisms, and performance regulation laws for major wrought and casting aluminum alloys. It also compares the characteristics of different alloy systems in terms of strength, ductility, corrosion resistance, fatigue performance, and manufacturing stability. The analysis shows that the development focus for traditional aluminum alloys has shifted from single performance improvement to multi-performance balance and precise control of the microstructure. With the increase in the proportion of recycled raw materials, the importance of impurity tolerance and the controllable transformation of second phases has further increased. At the same time, CALPHAD, physical models, and data-driven methods provide new means for alloy composition and process optimization, but their effectiveness still needs to be verified in actual components and engineering conditions. This paper can provide a reference for microstructure design, material selection, and performance optimization for the circular manufacturing of traditional aluminum alloys.

## 1. Introduction

Aluminum alloys are widely used structural materials for lightweight engineering. They offer high specific strength and stiffness, together with good formability, corrosion resistance, and recyclability. Over the past century, aluminum alloys have evolved from simple binary alloys to complex multi-component microalloying systems. They have also shifted from empirical heat treatment to the precise control of precipitate phases and grain boundaries. Thus, they have become fundamental materials in the aerospace, automotive, rail transit, shipbuilding, construction, and packaging industries [1,2,3]. Recent studies indicate that the development of high-strength aerospace aluminum alloys has shifted from merely enhancing strength to integrated design encompassing formability, microstructures, and service performance [4]. Similarly, research on low-cost aluminum alloys for high-temperature applications now places a greater emphasis on the synergistic effects of thermally stable precipitates, dispersed phases, and microalloying [5]. In advanced equipment, material selection has transitioned from an early focus on static strength to the balanced optimization of strength, ductility, fracture toughness, fatigue life, corrosion resistance, thermal stability, formability, and manufacturing costs [6,7,8]. Therefore, “traditional” does not imply fixed or stagnant material properties but rather reflects a mature industrial foundation, where further refinement in microstructure design and processing windows remains possible.

From the manufacturing perspective, industrial aluminum alloys can be classified into two main categories: wrought aluminum alloys and cast aluminum alloys. Wrought aluminum alloys are produced by further processing from ingots or continuous casting billets through rolling, extrusion, drawing, or forging. Plastic deformation not only alters the geometric shape of the product but also reconfigures the microstructure through dislocation proliferation, recovery, recrystallization, and texture evolution. The core of heat-treatable strengthening alloys such as 2xxx, 6xxx, and 7xxx lies in the evolution of supersaturated solid solutions and nano-sized precipitates, while 3xxx, 5xxx, and some 8xxx alloys rely more on solid solution strengthening, work hardening, and dispersion phase control. In contrast, cast aluminum alloys are directly formed into near-final components through liquid solidification, and their final performance is more constrained by factors such as the dendrite scale, the eutectic structure, intermetallic compounds, microsegregation, porosity, and oxide films. The differences between these two manufacturing paths determine that wrought alloys and cast alloys require the adoption of different microstructural optimization strategies.

The advantages of wrought aluminum alloys lie in their high structural integrity, the relatively low dispersion among their mechanical properties, and the ability to further adjust the grain structure, texture, and precipitated phases through thermomechanical processing. However, their limitations include the fact that complex-shaped components often require more forming and joining processes, and large-sized plates and forgings are also affected by the quenching speed, residual stress, and the gradient of the cross-sectional structure. Cast aluminum alloys have the prominent advantage of being able to achieve the one-time or few-time near-net shape forming of complex structures, and they are an important material foundation for automotive integrated structures and complex shells. However, fatigue and ductility are more likely to lead to critical pores, double oxide films, and local brittle phases. Therefore, there is no simple relationship in terms of performance superiority between the two types of alloys. Instead, the material application boundaries vary for different loading conditions, geometric complexities, service environments, production efficiencies, and forming cycles.

Sustainable manufacturing is further changing the design constraints of traditional aluminum alloys. Recycled aluminum significantly reduces the resources and energy input required for the production of primary aluminum, but the mixed waste aluminum introduces residual elements such as Fe, Si, and Cu, which are difficult to completely remove, presenting new challenges to alloy design that previously relied on high-purity raw materials and strict upper limits on impurities [9,10]. Kotadia et al. [11] pointed out that the type, size, and morphology control of Fe-enriched intermetallic compounds have become a key aspect in improving the impurity tolerance of recycled aluminum. This has shifted the research focus from simply reducing the impurity content to designing for impurity tolerance by regulating elements such as Mn and Cr and controlling solidification and heat treatment, thereby converting unavoidable residual elements into second phases with smaller sizes, less harmful morphologies, or potential strengthening effects. Marioara et al. [12] further studied the recycled-friendly 6082 and 6005 alloys and demonstrated that, after increasing the content of Fe, Cu, and Zn, it is necessary to coordinate the regulation of coarse second phases and precipitation behavior to maintain a stable microstructure and properties. Mrówka-Nowotnik et al. [13] also confirmed, through a study of recycled 2017A alloy, that an effective precipitation strengthening response can still be established through reasonable recycling and post-treatment. At the same time, short-flow casting, high-recovery-ratio die casting, and closed-loop automotive aluminum materials require the grade system to have stronger adaptability to raw material fluctuations [10,14].

Most existing reviews on aluminum alloys primarily focus on specific material systems, such as high-strength aerospace-grade aluminum alloys, 6xxx-series sheet alloys for automotive applications, and Al-Si casting alloys. Cross-system comparative studies on microstructure control methods and the performance trade-offs across different aluminum alloy systems remain relatively scarce. The traditional aluminum alloys discussed in this review mainly contain various mature commercial grades, including wrought aluminum alloys from series 2xxx to 8xxx, as well as mainstream casting alloys produced via conventional thermal–mechanical processing and casting techniques, such as Al-Si, Al-Cu, Al-Mg, and Al-Zn-Mg-Cu alloys. Based on a comprehensive logical framework linking the composition, processing, microstructure, properties, and service performance, this paper presents four innovative research contributions: first, a unified comparison of the characteristics and underlying principles between wrought and casting aluminum alloy systems; second, a consistent interpretation of the common mechanisms across different alloy systems using a standardized set of microstructural descriptors, including precipitate phases, grain boundary conditions, precipitate-free zones, dispersed phases, compositional segregation, and extreme defects; third, the integrated consideration of alloy impurity tolerance and the impact of fluctuations in recycled raw material properties during alloy selection and process optimization; fourth, a comprehensive analysis of phase diagram calculations and data-driven alloy design approaches, while also addressing scalability in production and practical engineering validation scenarios.

## 2. Cross-System Microstructural Fundamentals and Strengthening Mechanisms of Traditional Aluminum Alloys

The engineering properties of traditional aluminum alloys are the result of the combined effects of multiple microstructural scales. The main alloying elements determine the solubility of the matrix and the type of the main strengthening phase [15,16]; the microalloying elements control grain refinement, dispersion phases, and recrystallization [17,18,19]; and the residual elements may form coarse brittle phases or alter the solidification behavior. Recent systematic summaries of the nanoscale precipitation process in Al-Cu-Mg-Ag alloys further indicate that trace elements, interface structures, and precipitation pathways can jointly change the nucleation, stability, and strengthening efficiency of the precipitated phases [20]. Although different alloy series have their own typical grades, the underlying laws can be attributed to the redistribution of alloy elements during liquid solidification, hot processing, and aging, as well as the resulting interactions between grains, dislocations, precipitated phases, dispersion phases, and defects.

### 2.1. Alloying Elements, Second Phases, and Microstructure Formation

Cu, Mg, Si, and Zn are the most important primary alloying elements in traditional aluminum alloys. The high-temperature solubility of Cu in Al is significantly higher than its room-temperature solubility, so the Al-Cu and Al-Cu-Mg systems can form θ-type and S-type strengthening phases through solid solution, quenching, and aging [16,21]. Mg can mainly provide solid solution strengthening in 5xxx, form β″-type aging strengthening phases with Si in 6xxx [22,23,24], and also form η′- and η-type precipitated phases with Zn in 7xxx. Zhang et al. [25] showed that, in Al-Mg-Si alloys, precipitation hardening increases the strength while significantly altering the ductility and formability, indicating that the regulation of strengthening phases essentially requires balancing multiple objectives, such as strength, ductility, and formability. Si itself mainly contributes a low melting point and wear resistance functions in wrought 4xxx alloys and significantly improves fluidity and solidification behavior through a eutectic structure in cast Al-Si alloys. Its Mg-Si precipitated phase evolution in 6xxx is also affected by the specific phase structure [26]. Zn alone provides limited strengthening in Al, but, when combined with Mg and Cu, it can form a high-density aging precipitated system. Thus, the effect of the same alloying element needs to be analyzed comprehensively considering the specific phase balance relationship and processing path and cannot simply be attributed to a single increase in strength.

Although the addition amounts of elements such as Mn, Cr, Zr, Ti, and Sc are usually relatively low, they can significantly alter the microstructure stability. Ti often participates in the grain refinement of the as-cast microstructure through Al-Ti-B-type refining agents [17,19]. Zheng et al. [27] provided a systematic summary of the research on grain refinement in aluminum alloys, further indicating that the efficacy of nucleation particles and the solute-limiting growth effect must be considered together, and the refining effect is not determined solely by the addition amount of a single refining agent. Zr and Sc can form Al_3_Zr, Al_3_Sc, or composite dispersion phases with an L1_2_ structure, exerting a strong anchoring effect on subgrain boundaries and recrystallization interfaces [18]. Mn is both a main alloying element in 3xxx alloys and can form Al(Mn,Fe)Si dispersion phases with Fe and Si. It can also change the morphology of the Fe-rich phase in cast recycled aluminum [28]. Similarly to Mn, Cr can reduce recrystallization sensitivity by stabilizing the dispersion phase. The common feature of microalloying is that the optimal content range is narrow, and excessive addition is likely to form coarse primary intermetallic compounds, converting the strengthening benefit into plasticity loss and fatigue failure.

Fe and Si are the most typical residual elements in the recycling aluminum system. The equilibrium solubility of Fe in aluminum is very low, and it is prone to the formation of Fe-rich intermetallic compounds during solidification. For thin plates and foils, coarse Fe phases can affect rolling strip breakage, pinholes, and surface quality. For cast Al-Si, lamellar or needle-like Fe-rich phases can cause stress concentration and may form composite defects with pores. The traditional strategy emphasizes reducing the Fe content, but a more realistic approach in high-recycling-rate raw materials is to regulate the ratio of Fe to Mn and Si, increase the cooling rate, and optimize the heat treatment to make the morphology of the Fe-rich phase more compact. This idea provides important support for the transformation of traditional aluminum alloys from high-purity design to impurity tolerance design concepts [10,28,29]. In summary, the major alloying elements, representative phases, and microstructural roles in conventional aluminum alloys are shown in Table 1.

### 2.2. Main Strengthening Mechanisms

Traditional aluminum alloys typically incorporate a combination of solid solution strengthening, work hardening, precipitation strengthening, dispersion strengthening, and fine grain strengthening.

Solid solution strengthening results from the lattice distortion caused by the difference in the size and elastic modulus of solute atoms, and it is particularly important for 5xxx Al-Mg alloys. Work hardening increases the dislocation density through plastic deformation and is the main path for non-heat-treatable strengthened alloys to achieve strength. Both of these mechanisms have continuous and stable strengthening characteristics, but, as the solid solution elements and cold deformation degree increase, the forming load, recovery tendency, and local corrosion behavior of the material will also change. Therefore, they cannot be infinitely superimposed [15,16].

Precipitation strengthening is the core mechanism for 2xxx, 6xxx, and 7xxx wrought aluminum alloys, as well as some Al-Si-Mg, Al-Cu, and Al-Zn-Mg-Cu casting alloys. Solution treatment and quenching establish supersaturated solid solutions, followed by the evolution of atomic clusters, GP zones, and metastable precipitated phases in a specific sequence [21,22,23]. Fine coherent precipitated phases can be cut through by dislocations, and, as their size increases, they gradually shift to the bypass mechanism. The peak strength typically corresponds to the optimal combination of precipitate phase size and number density, rather than the state with the maximum precipitate volume fraction. Therefore, the process model proposed by Shercliff and Ashby [15], as well as subsequent atomic-scale studies, indicates that precipitation strengthening should be regarded as a coupled problem of phase transformation kinetics and dislocation interaction.

Dispersion strengthening and precipitation strengthening differ significantly in particle size and formation path. Dispersoids are usually formed during homogenization or high-temperature heat treatment, have relatively high thermal stability, and mainly hinder the motion of dislocations and the migration of grain and recrystallization boundaries. In contrast, low-temperature precipitates typically rely on a high number density to provide strengthening. Al_3_Zr and Al(Mn,Fe)Si are typical dispersoids. Dispersoids are particularly important for large-sized plates and extruded parts because they not only enhance the strength but also control the recrystallization fraction and grain morphology, thereby affecting anisotropy, fatigue, and quenching sensitivity [18,28].

Fine grain strengthening is of great significance in both cast and wrought alloys. The grain refinement during the solidification stage depends on the combined effect of heterogeneous nucleation particles and solute-limited growth. Interdependence Theory emphasizes that the number of effective nucleation particles and the constitutional undercooling caused by solutes must be considered in a coordinated manner [17,19]. In wrought alloys, the fine grain structure is also controlled by thermomechanical processing and recrystallization. It should be noted that finer grains do not always lead to better performance in all aspects. For example, an excessively high recrystallization ratio in high-strength 7xxx may reduce the stress corrosion performance, while some high-temperature applications require stable subgrains rather than completely equiaxed fine grains. Therefore, the grain size must be optimized together with the grain boundary precipitation state and the service mode.

### 2.3. Relationship Between Heat Treatment and Microstructure Properties

Heat treatment is a crucial step in transforming the chemical compositions of traditional aluminum alloys into controllable microstructures. For heat-treatable wrought alloys, the solution temperature needs to be high enough to dissolve the strengthening elements but must also be lower than the incipient melting temperature of the local low-melting-point phase. The quenching speed determines supersaturation and whether coarse precipitates occur during the cooling process. Artificial aging further controls the precipitation of clusters, metastable phases, and grain boundaries. For cast alloys, heat treatment also needs to consider the as-cast segregation, eutectic phase spheroidization, and pore expansion. Therefore, the process window for these alloys is usually more dependent on the wall thickness and initial defect level than for uniform-component thin plates.

Although non-heat-treatable alloys cannot achieve precipitation strengthening through solution treatment and aging, they still cannot be used without heat treatment. The homogenization of 3xxx determines the size and spatial distribution of the Al(Mn,Fe)Si dispersoids, thereby controlling the recrystallization and recovery during subsequent rolling. In 5xxx, β-Al_3_Mg_2_ may precipitate along grain boundaries during long-term exposure at medium temperatures, so the heat history itself is an important component of the service state. Meanwhile, 8xxx foil materials are regulated by homogenization, cold rolling, and annealing to adjust the Fe-rich phases, grains, and texture. Thus, it can be seen that heat treatment is not only solution treatment and aging but also includes the design of the microstructural evolution path of homogenization, cooling, aging, recrystallization, and stabilization.

The heat treatment of traditional aluminum alloys is a continuous process beginning with microstructure evolution; progressing through dissolution or homogenization, cooling, and precipitation or stabilization; and ultimately entering the service evolution stage. For heat-treatable wrought alloys, the focus is on obtaining high-density nano-precipitates and controlling grain boundary phases. For cast alloys, the focus is on reducing segregation, spheroidizing eutectic constituents, and enabling age hardening without inducing local melting and pore expansion. The final performance is not only determined by the microstructure at a certain moment but also depends on the stability of this microstructure during welding, painting, marine exposure, or medium-temperature service processes.

From an engineering perspective, it is necessary to weigh the microstructural advantages brought about by extending homogenization, solid solution treatment, or multi-step aging against the resulting thermal effects and energy consumption costs. The minimum sensible heat required to heat a batch of materials to the desired temperature is roughly proportional to Q_heat_ ≈ m∫C_p_(T)dT, while the actual heat input in the furnace also includes heat loss, the holding time, atmosphere control, and repeated heating/cooling stages. Therefore, even when the distribution of precipitated phases, solute dissolution, or the enhancement of precipitation effects tend to be limited, longer or staged process cycles may still increase the energy consumption per unit product and reduce the production efficiency. This is particularly evident in the multi-step homogenization of 3xxx series alloys and the multi-stage solid solution treatment of copper-containing cast alloys. Thus, heat treatment optimization should consider microstructure improvement, the pore expansion risk, the cycle time, and the unit product energy consumption as interrelated engineering goals, rather than simply considering longer processing times as an advantage.

## 3. Wrought Aluminum Alloy Systems and Property Control

A common feature of wrought aluminum alloys is that they continue to undergo significant plastic deformation and thermomechanical processing after solidification. Rolling, extrusion, and forging simultaneously alter the dislocation density, grain morphology, recrystallization fraction, and texture, and these changes are coupled with subsequent aging. Therefore, the evaluation of wrought aluminum alloys cannot be based solely on the nominal composition or peak strength. It should also consider the product form, cross-sectional size, heat treatment state, welding process, and service environment. This section discusses wrought aluminum alloys in three categories based on the dominant strengthening mechanism and finally conducts a cross-system comprehensive comparison.

### 3.1. Precipitation-Strengthened Wrought Aluminum Alloys: 2xxx, 6xxx, and 7xxx

Precipitation-strengthened alloys are formed through a complete set of heat treatment processes including solution treatment, quenching, and aging. They possess high-density nano-sized precipitated phases and have the best strengthening potential among traditional aluminum alloys. They are core load-bearing materials in the aerospace and high-end equipment fields.

#### 3.1.1. 2xxx Al-Cu-Mg High-Strength Aluminum Alloy

2xxx is one of the earliest aluminum alloy systems to achieve industrial age hardening. After Cu is dissolved at high temperatures and then quenched, a supersaturated solid solution is formed, followed by the nucleation and evolution of clusters, GPB zones, and θ-type and S-type precipitates. In Al-Cu-Mg alloys such as 2024, S-Al_2_CuMg and its metastable precursors significantly contribute to the strength [30,31,32], while, in high-Cu alloys, θ″ and θ′-Al_2_Cu are equally important [33,34]. Compared with 6xxx, the alloying degree and precipitation strengthening potential of 2xxx are higher, thus enabling the attainment of higher strength and better damage tolerance, but the coarse Cu-rich particles and electrochemical inhomogeneity at the grain boundaries are also more obvious.

**Figure 1:**
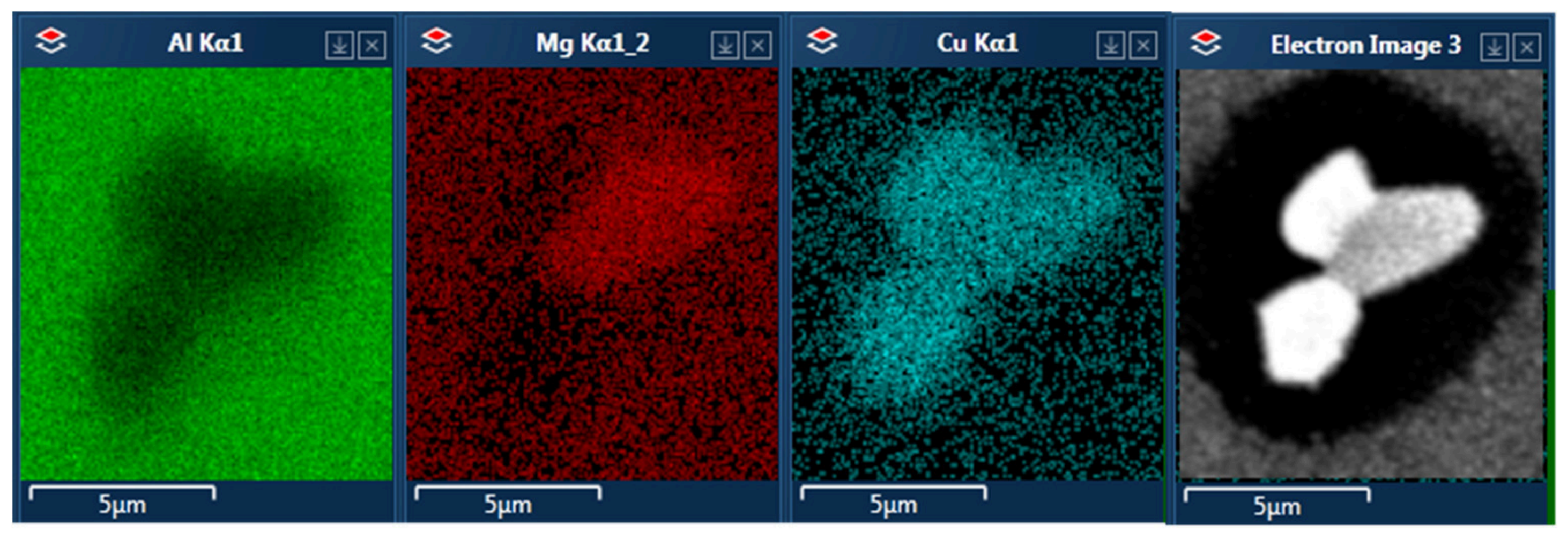
Representative Al_2_Cu and Al_2_CuMg phases in 2024 aluminum alloy [33].

The 2xxx structure exhibits distinct multi-scale characteristics. The nano-sized precipitated phases are responsible for aging strengthening, while the dispersed phases formed by elements such as Mn inhibit recrystallization. The coarse Cu and Fe particles remaining from the casting stage may serve as sources of fatigue and corrosion cracks. Staszczyk et al. [33] conducted the systematic characterization of the second phases in 2024 aluminum alloy under different heat treatment conditions. The results showed that the coarse crystalline phase, the dispersed phase, and the aging-precipitated phase exhibited significant differences in scale, morphology, and distribution, as shown in Figure 1. Atom probe studies further revealed that Cu and Mg had already undergone significant segregation in the early stage of aging [30,31], indicating that the establishment of the peak strength had already begun before the observable coarse precipitates appeared.

In addition to the coarse-grained component particles, the grain boundary-precipitated state also affects the corrosion behavior and stress corrosion cracking (SCC) susceptibility of 2xxx alloys. The solute redistribution caused by aging can produce grain boundary precipitates that are rich in Cu/Mg, accompanied by adjacent PFZs. When the grain boundary precipitates are sufficiently continuous, the resulting electrochemical and local strength gradients will promote intergranular preferential dissolution and strain localization. Therefore, the width of the PFZ should not be interpreted alone. Its influence on SCC depends on the continuity of the grain boundary precipitates, their chemical composition, the potential difference between the matrix and the precipitated phase, and the applied stress state [6,7,8,34].

Among the typical grades, 2024 and 2124 are commonly used for thin plates, fuselage skins, and damage-tolerant structures, while 2219 is employed for aerospace tanks and other structures due to its welding performance and medium-temperature stability. Compared to 7xxx, the advantages of 2xxx do not lie in its peak strength but in the damage tolerance, medium-temperature performance, and welding compatibility of some grades. Its main drawback is corrosion resistance and the synergy of strength and toughness. Therefore, modern 2xxx designs no longer simply increase the Cu content but instead control the precipitation of phases within and at the grain boundaries through high-purity, micro-alloying, pre-deformation, and multi-stage aging and reduce the harm of coarse particles.

For thick plates and complex forgings, the use of 2xxx also needs to address the issues of recrystallization and residual stress. A high recrystallization fraction may alter the continuity of grain boundaries and anisotropy, while excessive quenching stress can affect the dimensional stability during machining. Therefore, the nanoparticle precipitation design at the material end must be coordinated with quenching, tensile stress relief, and the processing route at the product end. Its most significant engineering feature is the mature balance of high strength, fatigue resistance, and damage tolerance required for aerospace applications.

#### 3.1.2. 6xxx Al-Mg-Si Composite Performance Aluminum Alloy

6xxx is one of the most important heat-treatable strengthened aluminum alloys, used in automotive, rail transportation, construction, and general extrusion structures. Its typical precipitation process encompasses stages such as supersaturated solid solution, solute clusters, and GP zones [22,23], including β″, β′, and β [24,26], among which the fine needle-like β″ usually bears the main strengthening effect at peak aging [35,36,37]. The early cluster and vacancy states determine the subsequent β″ nucleation efficiency [38,39], so natural aging, pre-aging, and artificial aging are not independent of each other but a continuous kinetic process [40].

Automobile sheet materials are particularly sensitive to this coupling. After solution quenching, the materials need to undergo storage and stamping, and then undergo a short paint-bake cycle. If natural aging forms clusters that are unfavorable for subsequent β″ nucleation, the bake hardening response will decrease. Therefore, body panels such as 6016 and 6111 require pre-aging to stabilize beneficial atomic clusters [38,39]. Structural extrusion materials require more attention to quenching sensitivity and section uniformity. When the wall thickness of the profiles increases or the online quenching speed is insufficient, coarse precipitates may occur during the cooling process and consume Mg and Si, resulting in a reduction in the subsequent aging potential [41].

**Figure 2:**
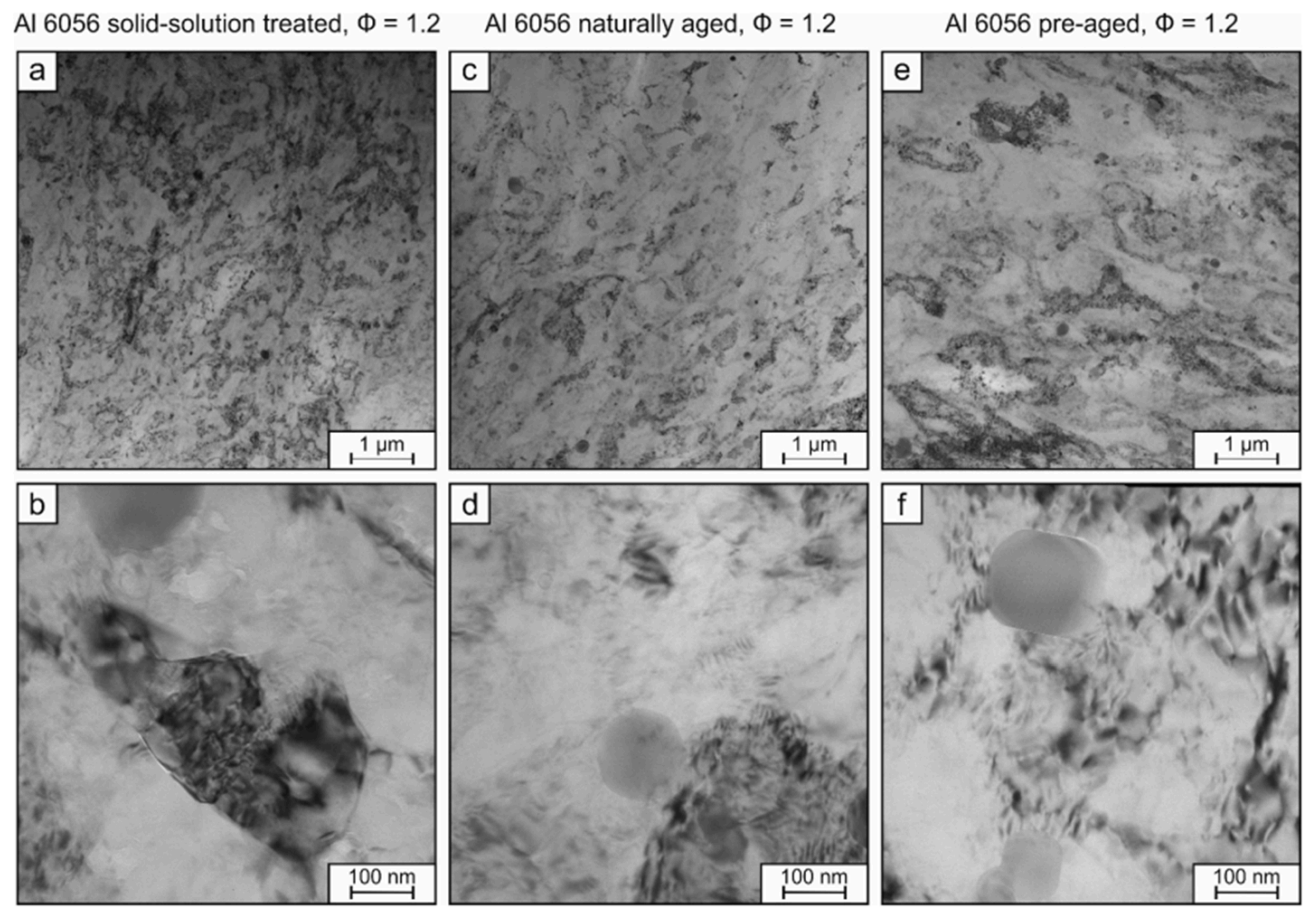
Precipitation microstructures of 6056 aluminum alloy under different aging histories: (**a**,**b**) solution-treated; (**c**,**d**) naturally aged; (**e**,**f**) pre-aged [42].

Winter et al. [42] compared the precipitated microstructure of 6056 aluminum alloy after solution treatment, natural aging, and pre-aging followed by artificial aging. The results showed that pre-aging could significantly alter the nucleation and distribution of the subsequent precipitated phases, as shown in Figure 2. The strengthening ability of 6xxx essentially stems from the precise utilization of the aging path. Peak aging is not the only engineering state; appropriate over-aging can improve the dimensional stability and performance reliability in certain environments. The effective quantity of β″ is determined jointly by the Mg-Si ratio, excess Si, Cu addition, the vacancy concentration, the quenching rate, and the pre-aging regime. Compared with the highly alloyed 7xxx, the composition window of 6xxx is wider and the manufacturing consistency is higher, which are key for its large-scale entry into automobiles and rail transportation.

Meanwhile, 6061 and 6082 represent medium-strength structural-grade 6xxx alloys, which have excellent extrusion properties, corrosion resistance, welding properties, and anodizing performance. For automotive exterior panels, higher formability, surface quality, and short-term bake hardening capabilities are required. Compared with 5xxx, the yield strength of 6xxx can be significantly increased through T6 aging, and its surface treatment performance is better, but the softening of the welding heat-affected zone is more obvious. Compared with 2xxx, 6xxx has lower strength, but it has better corrosion resistance, extrusion, and cost advantages.

For 6xxx, the factor that truly restricts its further application is not the peak aging strength but the performance fluctuations caused by the natural aging history and cross-sectional variations in the quench rate. Especially under the conditions of gradually increasing closed-loop recycling in the automotive industry, the influence of residual elements such as Fe and Cu on the precipitation process and surface quality merits re-evaluation.

#### 3.1.3. 7xxx Al-Zn-Mg-Cu Ultra-High-Strength Aluminum Alloy

7xxx is the system with the highest strengthening potential among traditional industrial wrought aluminum alloys. After solid solution and quenching, Zn, Mg, and Cu form a highly supersaturated solid solution, followed by the appearance of GP zones and η′- and η-MgZn_2_-type precipitated phases. Atomic-scale studies [43,44] have shown that the type of GP zone, the degree of Zn and Mg aggregation, and the vacancy state will affect the nucleation and growth of η′. High-strength grades such as 7050, 7055, and 7085 also inhibit recrystallization through Zr dispersion phases and reduce the sensitivity of thick sections to quenching. A characterization study of the precipitation process of 7055 further indicates the influence of the heat treatment state on the precipitation characteristics [45].

Compared with 2xxx, 7xxx is more capable of achieving industrial strength levels of 600 MPa or higher. However, high alloying simultaneously increases the sensitivity to quenching, stress corrosion, and the difficulty in controlling residual stress. The optimization of its microstructure must address nanoscale precipitation, grain boundaries, precipitate-free zones (PFZs), dispersion phases, and recrystallized grains. Simply increasing Zn and Mg usually cannot continuously enhance the engineering performance, as the quenching speed at the center of thick plates and the grain boundary state become new limiting factors.

**Figure 3:**
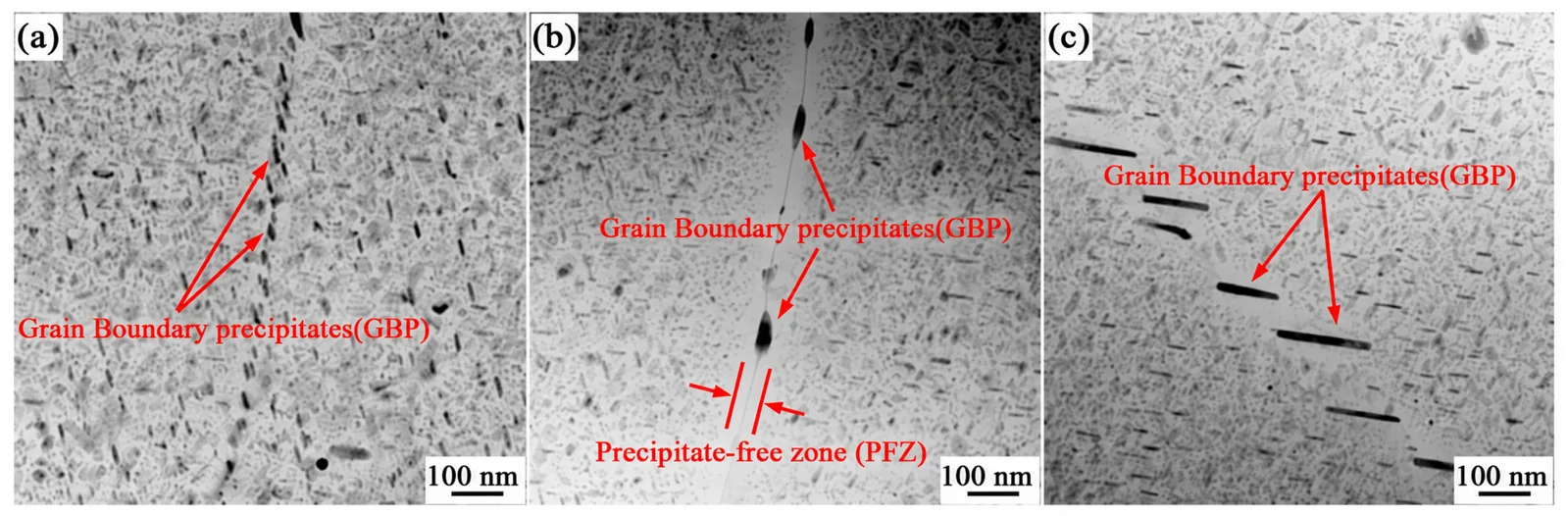
Characteristic grain boundary precipitation in 7055 aluminum alloy: (**a**) continuous boundary precipitates; (**b**) precipitate-free zone; (**c**) heterogeneous boundary precipitation [46].

The T6 peak aging state may lead to the precipitation of high-density fine intracrystalline inclusions, but the continuous grain boundary inclusions and adjacent PFZs will form electrochemical and local strength gradients, thereby promoting intergranular corrosion and strain localization. The T7 over-aged state improves the corrosion resistance and damage resistance by coarsening grain boundary inclusions and reducing their continuity, although it sacrifices some strength [46]. Li et al. [46] characterized the grain boundary inclusions and PFZs after the multi-stage solution aging of the 7055 alloy, and the results showed that there were differences between continuous boundary inclusions, non-included zones, and strip-like inclusions, as shown in Figure 3. Retrogression and re-aging (RRA) aims to restore the intracrystalline strengthening effect while maintaining the improved grain boundary state. Studies have shown that the RRA of AA7050 and AA7150 can effectively balance strength and SCC [47]. Therefore, the SCC resistance depends on the continuity and chemical composition of the grain boundary inclusions and their adjacent PFZ regions, rather than just the width of the PFZ.

The different heat treatment states of 7xxx essentially involve choosing different positions in the multi-performance space. T6 is closer to the strength end, T7 is closer to the corrosion resistance and damage tolerance end, and RRA is in between the two. The optimal state of the actual product is also influenced by the thickness, residual stress, surface treatment, and service environment. Thus, it can be seen that the development focus of 7xxx has shifted from achieving higher peak strength to the multi-objective optimization of strength, fracture toughness, fatigue, corrosion resistance, and manufacturing stability.

In large-sized thick plates and forgings, the quenching sensitivity determines whether the surface and the center can achieve similar supersaturation. Nie et al. [48] compared 7050 and 7085, indicating that the difference in composition would significantly alter the coarse precipitate behavior and quenching sensitivity during continuous cooling. The core value of low-quenching-sensitivity grades such as 7085 lies in maintaining higher strength and toughness and uniform corrosion resistance in thick sections.

Residual stress is also an important engineering constraint for large-sized 7xxx materials. Rapid quenching helps to retain solutes but results in higher thermal stress and deformation risks. Stretching for stress relief, compression pre-deformation, and optimizing the quenching medium need to be combined with subsequent aging for design. Future 7xxx research and development should clearly distinguish between small-sized laboratory specimens and industrial thick plates. Any ultra-high strength data should be reported simultaneously with the component thickness, quenching conditions, and corrosion resistance evaluation.

### 3.2. Solid Solution Strengthening, Work Hardening, and Dispersion Phase-Controlled Alloys: 3xxx and 5xxx

This type of alloy cannot be significantly strengthened through aging. Its performance mainly relies on solute solid solution, work hardening, and dispersion phase regulation at high temperatures. The prominent advantage lies in the balance of formability, corrosion resistance, and cost.

#### 3.2.1. 3xxx Al-Mn Alloy

**Figure 4:**
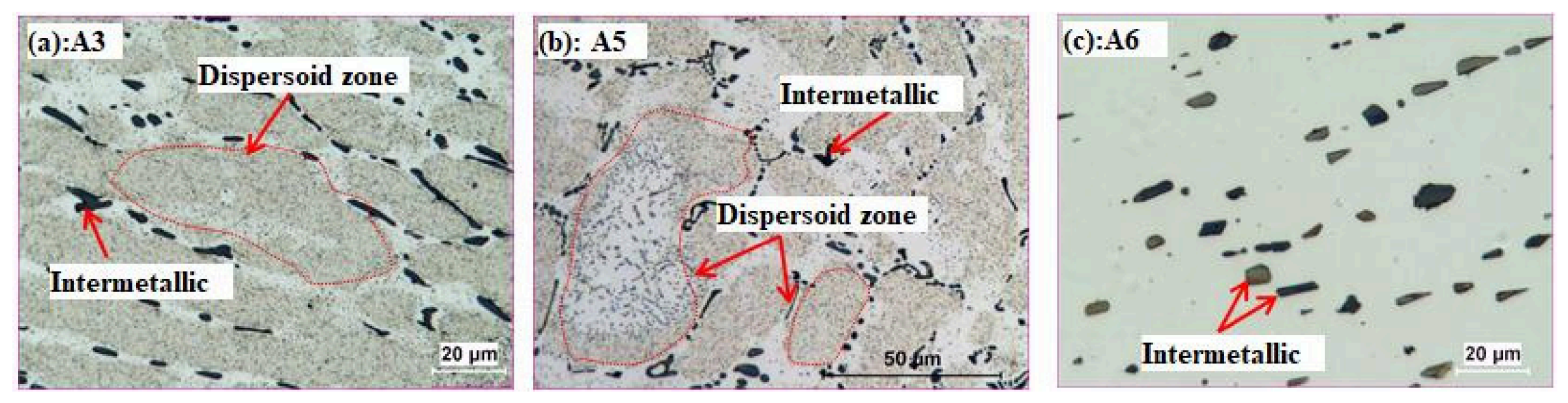
Dispersoid distributions in a 3xxx aluminum alloy after different single-step heat treatments [49].

3xxx is an alloy with Mn as the main alloying element and is a typical non-heat-treatable wrought aluminum alloy. The solubility of Mn in Al is limited, and, during homogenization and multi-step heat treatment processes, an Al(Mn,Fe)Si dispersoid is formed. These dispersoids not only provide direct strengthening but also inhibit high-temperature recovery and recrystallization by pinning subgrain boundaries [49,50,51]. Wang et al. [49] compared the distribution of dispersoids under different single-step heat treatment conditions and found that the heat treatment path could significantly change the number and spatial distribution of the dispersoids, as shown in Figure 4. Unlike the low-temperature aging strengthening of 6xxx and 7xxx, the heat treatment of 3xxx focuses more on the formation of dispersoids and their control over recovery and recrystallization [52].

The room-temperature strength limit of 3xxx is significantly lower than those of 2xxx, 6xxx, and 7xxx, but its formability, corrosion resistance, weldability, and cost combination have long-term advantages. In particular, 3003 and 3004 are widely used in heat exchange, packaging, and construction products. Multi-step homogenization can increase the number of dispersed phases and change the creep threshold stress, indicating that low-strength alloys also have significant microstructural engineering potential [49,50]. Under the condition of a high proportion of recycled raw materials, fluctuations in Fe and Si will further change the dispersed phases and coarse intermetallic compounds, so the ratios of Mn, Fe, and Si and a uniform heating path will become important control parameters for the quality stability of 3xxx.

#### 3.2.2. 5xxx Al-Mg Corrosion-Resistant Aluminum Alloy

5xxx is mainly strengthened by Mg solid solution and work hardening. When Mg enters α-Al, it causes significant lattice distortion, increasing the resistance to dislocation movement, while maintaining a high work-hardening capacity and low-temperature toughness [53,54]. In particular, 5052 is mainly focused on forming and corrosion resistance, while 5083 and 5086 and other higher Mg grades have both higher strength, weldability, and marine environment adaptability. Compared with 3xxx, 5xxx has higher strength but also higher processing loads and material costs. Compared with 6xxx, 5xxx can obtain stable medium strength without relying on aging, and the performance changes after welding are easier to control, but it cannot obtain significant peak strengthening through artificial aging.

The most significant long-term service risk for 5xxx is sensitization [55,56,57]. Exposure to moderate temperatures for a long period promotes the precipitation of the β-Al_3_Mg_2_-related phase along high-angle grain boundaries. As the coverage of grain boundaries increases, the local electrochemical activity is significantly enhanced, and the intergranular corrosion and stress corrosion susceptibility subsequently increase [58,59,60]. Cold processing alters the dislocation density and diffusion conditions near the grain boundaries, thereby affecting the sensitization kinetics [61,62]. After components undergo welding, paint baking, or long-term warm service, even if the room-temperature strength still meets the requirements, the corrosion resistance state may have already changed.

**Figure 5:**
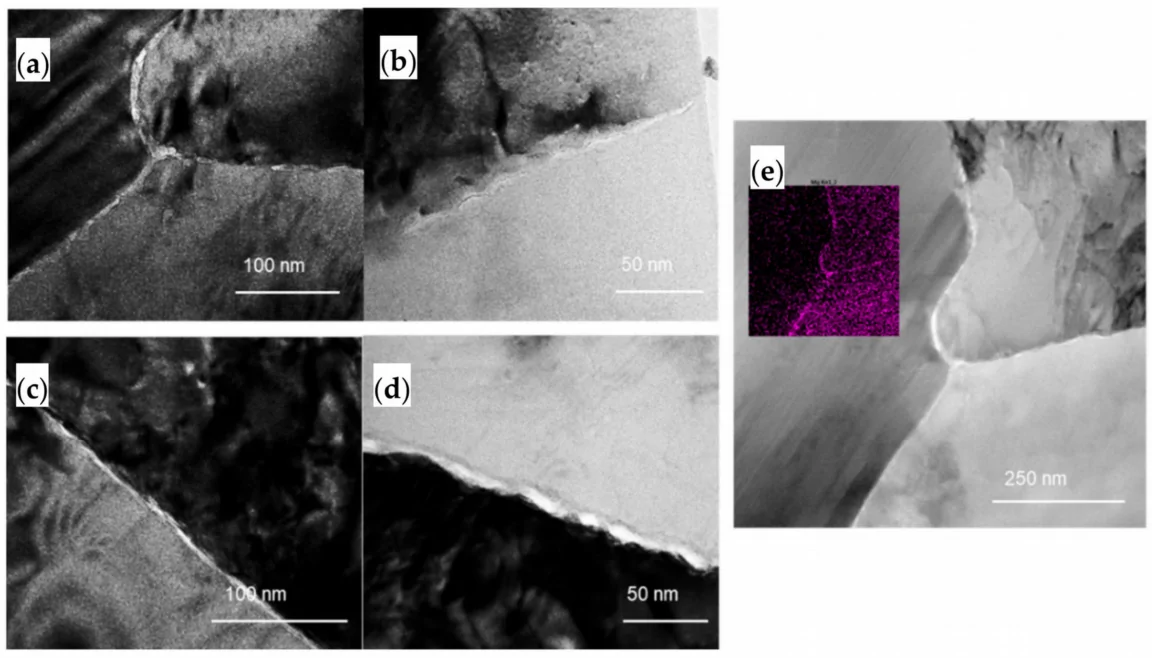
Evolution of grain-boundary β precipitates in Al-6Mg alloys subjected to different deformation and thermal exposure conditions: (**a**) CR20 alloy, 48 h; (**b**) CR50 alloy, 48 h; (**c**) CR20 alloy, 207 h; (**d**) CR50 alloy, 207 h; (**e**) TEM-EDS (Mg element) CR20 alloy, 48 h [62].

Choi et al. [62] conducted a study using TEM and elemental distribution analysis to investigate the evolution of grain-boundary β phases in Al-6Mg alloys under different exposure times and cold deformation conditions. The results showed that, during the sensitization process, the precipitated phases at the grain boundaries gradually increased and became continuous, as shown in Figure 5. The 5xxx alloy can transition from a low-sensitization state to a sensitization state where continuous grain-boundary β phases precipitate through medium-temperature exposure and then through appropriate regression or desensitization treatment to reduce the continuous coverage of grain boundaries. This process reveals the most typical performance contradiction of 5xxx—that is, Mg solid solution strengthening aims to maintain high solute content, while the long-term stability of grain boundaries requires the inhibition of the continuous precipitation of β phases. Therefore, when evaluating materials such as 5083 for ships, the thermal history, sensitization degree, and welding heat cycle should be included in the material state definition, rather than merely comparing the initial tensile strength.

The most distinctive feature of 5xxx is the combination of resistance to seawater corrosion, weldability, plasticity, and low-temperature toughness. For ships, low-temperature storage tanks, and welded transportation structures, this combination is often more valuable than the peak strength. Future optimization directions include reducing the sensitization kinetics, utilizing Mn and trace elements to stabilize substructures, controlling Fe and Cu in recycled raw materials, and establishing a life evaluation model corresponding to the actual temperature–time spectrum.

### 3.3. Function-Oriented 4xxx and Representative 8xxx Alloys

This type of alloy does not focus on structural load bearing as its core objective. Instead, it is designed to meet specific functional requirements such as joining, wear resistance, and ultra-thin forming.

#### 3.3.1. 4xxx Al-Si Alloy

4xxx is an alloy with Si as the main alloying element, and it has more functional material properties in wrought aluminum alloys. In particular, 4043 and 4047 are mainly used as welding filler wires and brazing materials. The Si is utilized to lower the melting point, improve the flow of the molten pool, and reduce the tendency for hot cracking. Alloys with higher Si content and containing Mg, Cu, and Ni, such as 4032, can achieve better wear resistance and high-temperature dimensional stability through heat treatment and Si particle refinement. In aluminum and steel welding and brazing, Si can also change the growth of intermetallic compound layers at the interface. Therefore, the core competitiveness of 4xxx lies in joining and wear resistance, rather than high load-bearing strength [63].

#### 3.3.2. Representative 8xxx Al-Fe-Si Foil Alloys

8xxx is not a single Al-Fe alloy series. This article only discusses the representative Al-Fe-Si foil material branches among them, such as 8011 and 8079. The equilibrium solubility of Fe in Al is extremely low. The Fe-rich intermetallic compounds formed during solidification and homogenization processes directly affect recrystallization, ultra-thin rolling, pinholes, and surface quality. An in situ TEM study of AA8079 indicates that the Fe-rich phase transformation during the homogenization process can change the subsequent cold rolling and annealing behavior [64]. Compared with 3xxx, 8xxx Al-Fe-Si is more suitable for ultra-thin foils and packaging, but it is more sensitive to the size of the second phase, segregation, and the rolling history.

### 3.4. Comprehensive Comparison of Wrought Alloys

There is no unified optimal series for different wrought aluminum alloys. The 2xxx series has advantages in terms of high strength, fatigue resistance, and some mid-temperature scenarios. The 3xxx series excels in low-cost forming and corrosion resistance. The 4xxx series fulfills the functions of joining and wear resistance. The 5xxx series is suitable for marine corrosion-resistant welding structures. The 6xxx series is the most balanced in terms of strength, forming, corrosion resistance, and mass production. The 7xxx series provides the highest specific strength. The 8xxx series, representing Al-Fe-Si, is oriented towards ultra-thin foil materials and packaging functions. Table 2 summarizes the relative performance and key bottlenecks of different systems.

Table 2 shows that, for different products, what truly has no substitute is often the comprehensive performance of the material throughout the entire process of manufacturing, joining, and service. For example, marine welding structures are more suitable applications for 5xxx; automotive large-scale paneling and extruded structures are more suitable scenarios for 6xxx; and high-strength aviation thick plates are the ideal scenario for 7xxx. The focus of future material design should be on addressing the most significant shortcomings based on the inherent advantages of each series in order to achieve the optimal matching of performance.

Importantly, the static tensile strength alone cannot fully reflect the actual service performance of wrought aluminum alloys. Fatigue performance should not be directly inferred from the tensile strength either. Usually, fatigue cracks originate from coarse grains, inclusions, surface defects, regions of uneven microstructure, grain boundary conditions, and local recrystallization, which all affect the subsequent crack propagation. This helps to explain why the peak strength conditions in the 2xxx and 7xxx series alloys do not always provide the best balance between fatigue and corrosion [6,7,8,33,46]. A quantitative comparison is also highly dependent on the annealing state and processing history. For example, a previous study on the 6056 alloy indicates that, when artificial aging treatment is performed after pre-aging and extrusion, its ultimate tensile strength increases from approximately 470 MPa to 532 MPa [42]. These values should be understood as reference values under specific process conditions, rather than universal ranking standards.

## 4. Control of Solidification Structure and Properties of Cast Aluminum Alloys

Compared with wrought aluminum alloys, many key structures of cast aluminum alloys are established during the solidification process. The α-Al grains and the spacing of secondary dendrite arms determine the matrix size; the eutectics and intermetallic compounds determine the distribution of local hard brittle phases; and hydrogen precipitation, solidification shrinkage, and oxide film entrainment form porosity and double oxide films. Subsequent heat treatment can modify the morphologies of eutectics and precipitated phases, but it cannot completely eliminate macroscopic segregation, large-sized pores, and entrained oxide films. Therefore, the performance optimization of cast alloys must start from the melt quality, solidification kinetics, and defect formation, rather than relying solely on the subsequent T6 treatment. Considering the dominant position of Al-Si alloys in industrial applications and their rich research foundation, this section places a greater emphasis on the discussion of this alloy system rather than other cast aluminum alloy systems.

### 4.1. Solidification Structure and Defect Characteristics of Cast Aluminum Alloys

The casting process initially involves heterogeneous nucleation and dendrite growth. The effective nucleation particles, constitutional undercooling, and the cooling rate jointly determine the number of α-Al grains [17,19]. Fine grains not only facilitate the improvement of microstructure uniformity but also shorten the inter-dendritic segregation scale. Subsequently, eutectics and intermetallic compounds form in the remaining liquid phase, and their morphology and continuity determine local stress concentration and the difficulty of subsequent solution treatment. Systematic studies have been conducted on the eutectic Si in Al-Si and its modification rules [65,66], while Al_2_Cu in Al-Cu and the Fe-rich phase in alloys containing Fe represent other typical solidification second phases [29].

For cast aluminum alloy components, the impact of casting defects on service performance is as crucial as the matrix structure. The solubility of hydrogen in liquid aluminum is much higher than that in solid aluminum. During solidification, hydrogen precipitation can form pores. Volume contraction and insufficient feeding can lead to porosity. The oxide film on the surface of the molten metal, when entrained during turbulent filling, forms a double oxide film. The sizes, shapes, and locations of pores and oxide films exhibit strong randomness, so the fatigue life of the castings often shows greater dispersion than in wrought products. For highly reliable castings, the maximum defect size and the defect distribution in high-stress areas are more critical performance evaluation criteria than the average porosity.

### 4.2. Al-Si Casting Alloys

Al-Si is one of the most widely used casting aluminum alloy systems in industrial applications [67,68,69]. Si can significantly improve fluidity and reduce solidification shrinkage and the tendency for hot cracking, making this system particularly suitable for thin-walled, complex, and large-scale integrated structures. Alloys such as A356 and A357, which combine casting properties with T6 strengthening, and Al-Si-Cu-Mg alloys of the 319 type, which enhance strength and thermal stability through Cu, and the hypereutectic A390, which achieves higher wear resistance by utilizing a high volume fraction of primary crystalline Si, all demonstrate the major advantage of a comprehensive balance among the casting window, complex forming, cost, and heat treatability.

#### 4.2.1. Grain Refinement and Eutectic Si Modification

In the unmodified Al-Si eutectic, Si often appears as coarse lamellar or needle-like structures and forms stress concentration under tensile and fatigue loads [67,70]. Elements such as Na and Sr can modify the nucleation and growth of the eutectic Si, transforming it into fine fibers or granular structures, thereby enhancing the ductility and fatigue resistance [71,72,73]. Sr has the characteristics of long-term effectiveness and stable industrial operation, but its effectiveness depends on the residual Sr content, the holding time of the melt, the cooling rate, and the coexisting elements such as Mg and Cu [74,75,76].

Modification is not improved indefinitely by increasing the level of modifier addition. An A356 study shows that an appropriate amount of Sr can significantly improve the morphology of the eutectic Si, and further microstructural refinement tends to reach saturation. Long-term holding will also lead to modification fading [70,73,77]. Rare earth elements can also affect the eutectic Si and primary crystalline Si, but, usually, a higher addition amount is required, and new intermetallic compounds may be formed [78,79]. Therefore, industrial modification requires the unified control of the addition amount, residual amount, holding time, and cooling rate.

**Figure 6:**
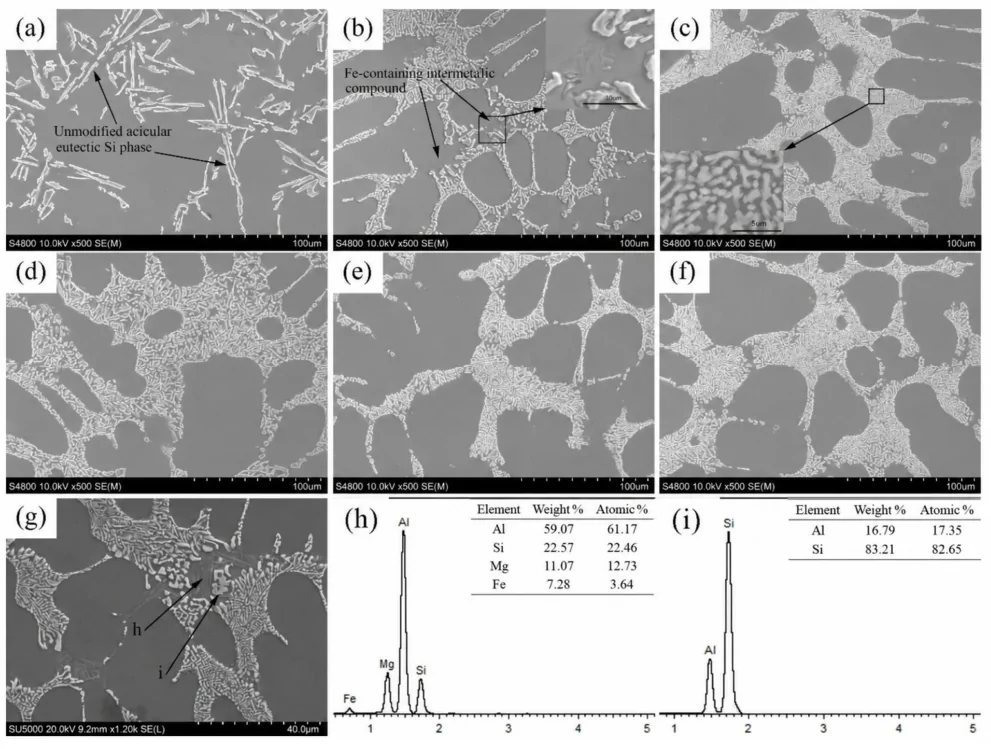
Effects of Sr addition on the morphologies of eutectic Si and Fe-rich phases in A356 alloy: (**a**) 0.00%; (**b**) 0.02%; (**c**) 0.03%; (**d**) 0.04%; (**e**) 0.05%; (**f**) 0.06%; (**g**) morphology of iron-rich phase and partially modified eutectic Si; (**h**,**i**) corresponding EDS results [70].

Zhang et al. [70] investigated the effects of different Sr addition amounts on the morphologies of the eutectic Si and Fe-rich phases in A356 alloy. The results showed that changes in Sr content significantly altered the refinement and fibrous morphology of the eutectic Si, as shown in Figure 6. The evolution of eutectic Si can be summarized as follows: coarse lamellar Si in the unmodified state, fine fibrous Si after appropriate modification, and an inhomogeneous microstructure after excessive addition or modification fading. This result emphasizes the synergy between the modifier and the solidification conditions, and Sr or rare earth elements cannot be regarded as separate strengthening elements [71,72]. For recycled Al-Si, residual elements such as Fe and P may also affect nucleation and the efficiency of the treatment [80,81], so the treatment window needs to be re-determined according to the raw material system [82,83]. 

#### 4.2.2. Heat Treatment and Phase Evolution

**Figure 7:**
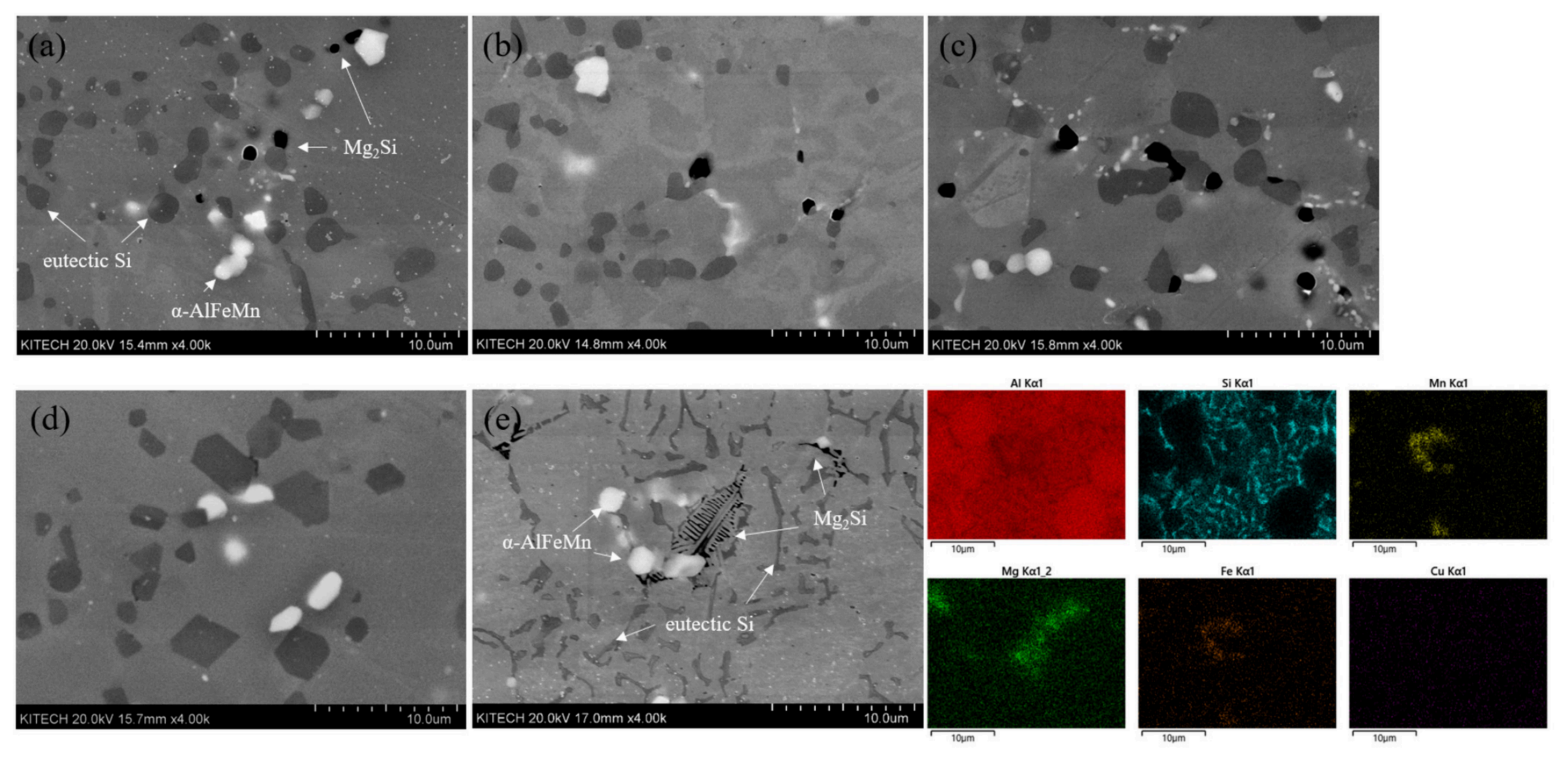
Microstructural evolution of a die-cast Al-Si-Mg alloy at different solution treatment temperatures (**a**) 480 °C; (**b**) 500 °C; (**c**) 520 °C; (**d**) 540 °C; (**e**) EDS images of the as-cast alloy [84].

The typical T6 process of Al-Si-Mg alloys causes Mg and Si to re-enter the α-Al matrix through solid solution, while promoting the fragmentation, rounding, and spheroidization of the eutectic Si. Subsequently, quenching and artificial aging form Mg-Si-type strengthening phases [68,84]. Kang et al. [84] compared the microstructure evolution of die-cast Al-Si-Mg alloys at different solution treatment temperatures, and the results showed that the solution treatment temperature directly affects the dissolution of the second phase, the morphology of the eutectic structure, and the subsequent aging potential, as shown in Figure 7. Al-Si-Cu-Mg further forms θ-type Al_2_Cu and Q-type complex phases, thus having higher strengthening and heat resistance potential, but with a narrower solution treatment window [85,86,87]. The local melting point of the Cu-rich interdendritic phases is lower, and an excessive solution treatment temperature will cause incipient melting and pore expansion, so multi-stage solution treatment has greater value [88,89,90].

The design goals of low-Cu Al-Si-Mg and high-Cu Al-Si-Cu-Mg are different. The former emphasizes toughness, corrosion stability, and structural integration and is suitable for automotive structural die casting and collision-resistant components. The latter enhances the peak strength, wear resistance, and medium-temperature performance through Cu and is more suitable for power components. For high-pressure die casting, heat treatment must also consider gas pore expansion; vacuum die casting and short-term, low-temperature heat treatment can broaden the dimensional stability window [84,89,91]. This indicates that the casting process determines whether the subsequent heat treatment can fully release the precipitation strengthening potential of the material.

#### 4.2.3. Fe-Rich Phases, Pores, and Fatigue Performance

Fe is an element that is difficult to completely remove from recycled aluminum. In Al-Si alloys, Fe-rich intermetallic compounds are often formed [29,83]. High-aspect-ratio plate-like or needle-like structures have a significant cutting effect on the matrix, while the addition of Mn, faster cooling, and appropriate composition adjustment can promote a more compact α-Al(Fe,Mn)Si-like morphology. Therefore, the design focus of recycled Al-Si is gradually shifting from strictly limiting Fe to controlling the type, size, and spatial distribution of the Fe-rich phase.

**Figure 8:**
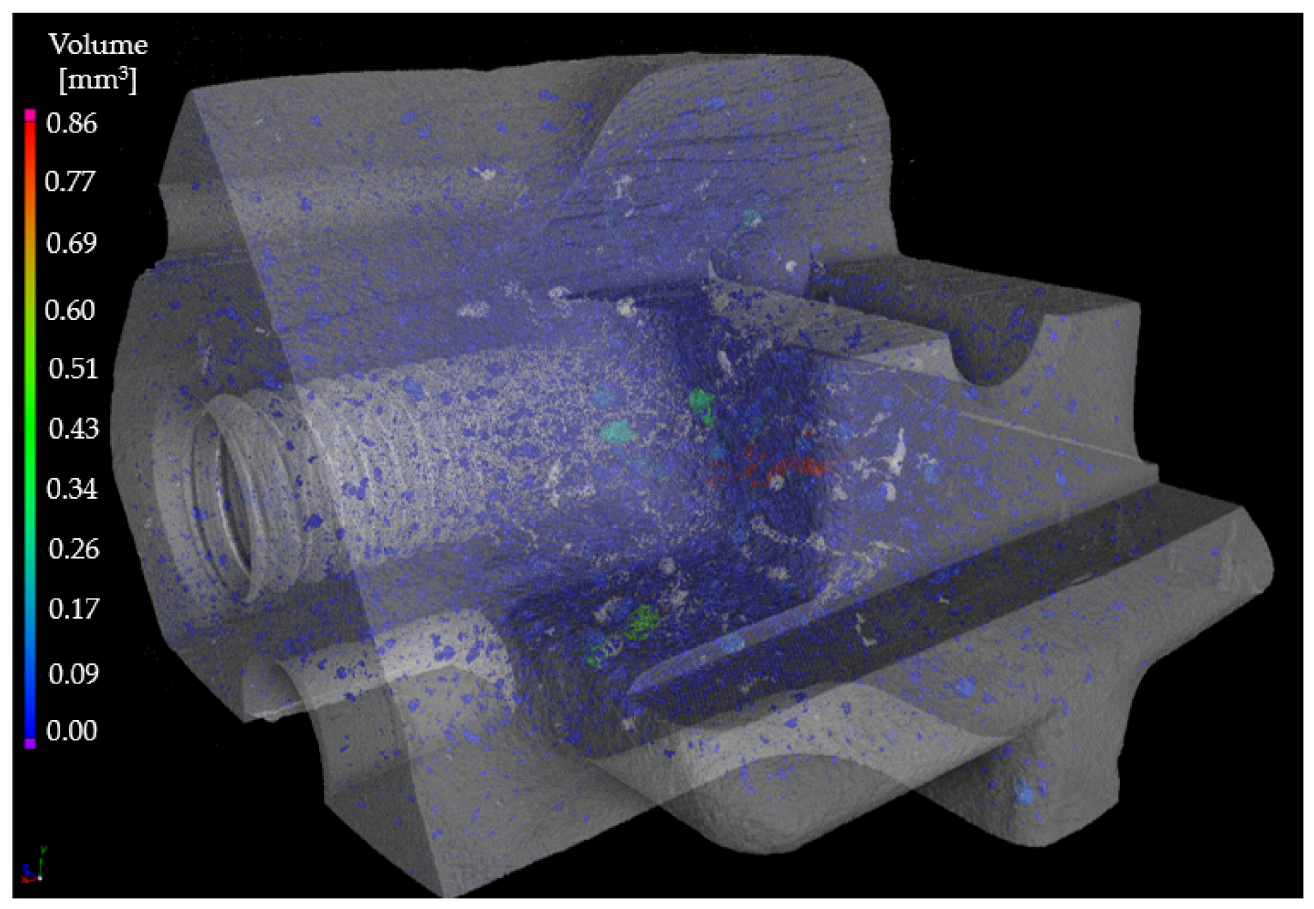
Three-dimensional CT reconstruction and spatial distribution of porosity in a die-cast aluminum alloy [93].

Porosity is another key factor affecting the fatigue reliability of Al-Si castings. Liu et al. [92] conducted research on the high-pressure die casting of Al-Si-Mn-Mg and pointed out that the characteristics of pores and eutectic particles jointly influence the tensile properties. From an engineering perspective, the maximum defect size and the distance from the defect to the free surface are usually more indicative of the risk of crack initiation than the average porosity rate. Réger et al. [93] used computed tomography to reconstruct the three-dimensional volume distribution of pores within die-cast aluminum alloys, providing an intuitive basis for identifying the connectivity of pore spaces and extreme defects, as shown in Figure 8. Future quality control should incorporate further XCT three-dimensional characterization and extreme statistical analysis.

The most significant engineering feature of the Al-Si system is the integration of casting performance and adaptability to complex structures. The main bottleneck is the performance dispersion caused by the coexistence of eutectic Si, Fe-rich phases, pores, and oxide films. With the development of vacuum high-pressure die casting and integrated body structures, low-defect molten alloys and heat-treatable die-cast alloys will continue to expand the structural application boundaries of Al-Si.

### 4.3. High-Strength and Heat-Resistant Al-Cu Cast Aluminum Alloys

Al-Cu is an important branch of conventional cast aluminum alloys in terms of high strength and heat resistance [94,95,96]. After solution treatment and quenching, Cu forms a supersaturated solid solution, and, during the aging process, θ″ and θ′-Al_2_Cu are formed; moreover, in alloys containing Mg, the S-type phase may also be formed [97]. Compared with Al-Si, Al-Cu has poorer fluidity and a wider solidification range, with greater difficulty in controlling cracking and shrinkage, but its aging strengthening and medium–high-temperature load-bearing potential are more prominent [98,99,100]. Therefore, this system is suitable for complex components that require high strength and heat stability and can accept more stringent casting process control.

During the later stage of solidification, Cu tends to accumulate between dendrites, forming coarse Al_2_Cu and low-melting-point eutectics. The solution treatment not only needs to fully dissolve these non-equilibrium phases and increase the supersaturation of the matrix but also must avoid local incipient melting. Therefore, the heat treatment window of Al-Cu alloys is usually narrower than that of Al-Si-Mg alloys. Multi-stage solution treatment and secondary aging can establish a more reasonable balance between phase dissolution, precipitation strengthening, and dimensional stability [97,100].

**Figure 9:**
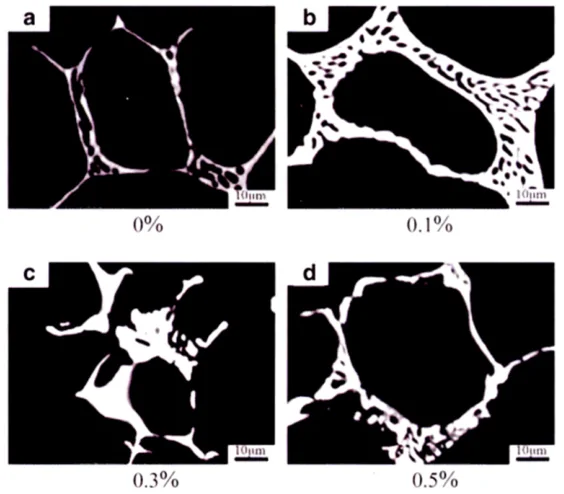
Representative microstructural and fracture morphologies of the Al-Cu-Mn alloy under different conditions: (**a**) as-cast Al–Cu–Mn alloy without Y addition; (**b**) alloy with 0.1 wt.% Y; (**c**) alloy with 0.3 wt.% Y; (**d**) alloy with 0.5 wt.% Y [94].

Trace elements such as Ti, Zr, Y, Er, Sm, and Cr can further enhance the performance by refining the cast microstructure, forming thermally stable dispersion phases, or altering the θ-phase nucleation kinetics [94,95,96]. Guo et al. [94] investigated the influence of trace Y addition on the fracture properties and microstructure of Al-Cu-Mn alloys, indicating that microalloying could change the morphology of the second phase and the crack propagation path, as shown in Figure 9. Among them, Ti and Zr can act on both the cast microstructure and the subsequent dispersion phases simultaneously, while Y, Er, Sm, etc., can modify the second phase and the fracture path [98,99,100]. Microalloying also has optimal content. Excessive addition will form coarse grain boundary compounds and damage plasticity, so it must be evaluated together with strength, elongation, and high-temperature stability [97].

The comparison between Al-Cu and Al-Si shows that the two systems cannot be ranked simply as high-strength and low-strength alloys. Al-Si has obvious advantages in terms of complex thin-walled filling, cost, and process tolerance. Al-Cu, on the other hand, has greater potential in terms of its high strength and intermediate-temperature load-bearing capabilities. Compared with Al-Mg, Al-Cu can obtain higher strength through aging, but its adaptability to corrosive environments is poorer. The core bottlenecks are solidification segregation, hot cracking, and a narrower solution range.

### 4.4. Al-Mg and Al-Zn-Mg-Cu Cast Aluminum Alloys

#### 4.4.1. Al-Mg Corrosion-Resistant Cast Aluminum Alloys

The Al-Mg cast alloy mainly relies on the solid solution strengthening of Mg in α-Al, resulting in good matrix continuity and outstanding resistance to atmospheric and seawater corrosion [101,102]. Compared with Al-Si, its melt fluidity is poorer, but it has advantages in plasticity, toughness, and surface quality. Compared with Al-Cu, its peak strength and high-temperature stability are lower, but it is more suitable for marine equipment, instrument shells, and corrosion-resistant components.

**Figure 10:**
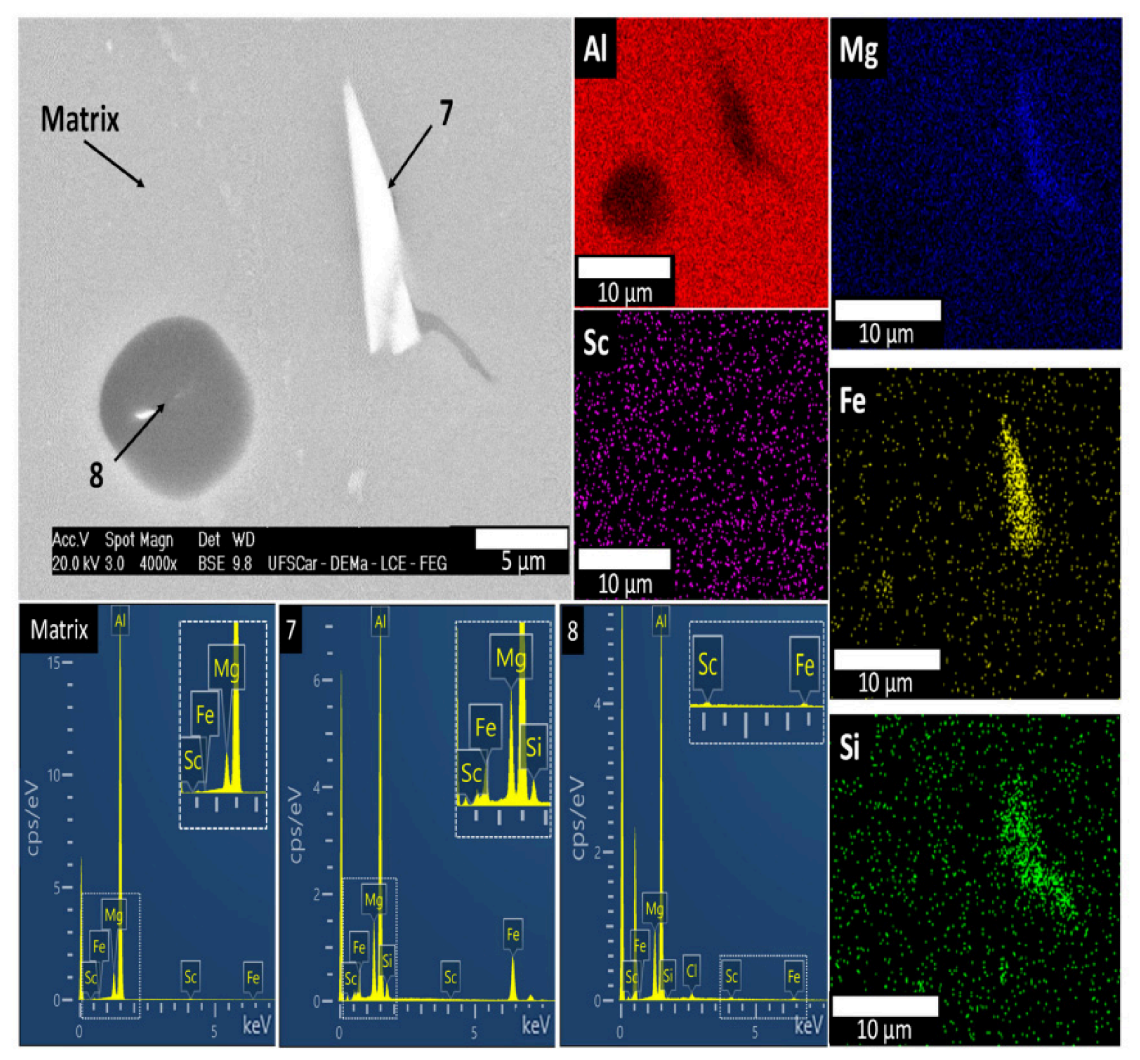
SEM-EDS characterization of constituent phases in the Al-5Mg-0.4Sc alloy solidified at a cooling rate of 1.3 °C/s [105].

Increasing the Mg content can enhance solid solution strengthening and significantly increase the risks of melt oxidation and inclusions. The surface oxide film of the high-Mg melt that is entrained during filling may form a hidden crack source. Therefore, melt protection, degassing, filtration, and low-turbulence filling are particularly important for Al-Mg. Sc, Zr, and Er can further refine the grains and form thermally stable dispersed phases, improving the microstructure stability. However, Sc is more costly, and industrial design needs to balance the material cost with the performance benefits [101,103,104]. The recent characterization of a cast Al-5Mg-0.4Sc alloy further revealed the distribution of Mg-, Sc-, Fe-, and Si-containing constituents in the as-cast microstructure through SEM-EDS analysis, as shown in Figure 10 [105].

Fe in recycled raw materials poses a representative challenge for the impurity-tolerant redesign of Al-Mg alloys. Studies have shown that, in specific Al-Mg and Al-Mg-Mn die-casting systems, Fe is not absolutely harmful. By regulating the Mn/Fe ratio, the Fe-rich intermetallic phase can be transformed into a more compact morphology, and a strengthening effect can be achieved under suitable conditions [106,107]. This indicates that, in the future, high-recycled-content Al-Mg alloys can be achieved by shifting from extremely low Fe to an acceptable Fe range with a controllable phase morphology, provided that reliable fatigue and corrosion verification is established.

#### 4.4.2. Al-Zn-Mg-Cu High-Strength Cast Aluminum Alloy

The Al-Zn-Mg-Cu casting system has attracted attention in recent years due to its high strength potential. The combination of Zn, Mg, and Cu can form η′- and η-type MgZn_2_-related strengthening phases during subsequent aging [108,109], enabling the as-cast material to achieve a basic precipitation mechanism similar to that of 7xxx wrought alloys [110,111]. The advantage is that it can be directly cast into complex high-strength components, but a limitation is the lack of sufficient plastic deformation to achieve densification and microstructure reconfiguration, thus resulting in the stronger effects of pores, dendrite segregation, and coarse secondary phases on performance.

**Figure 11:**
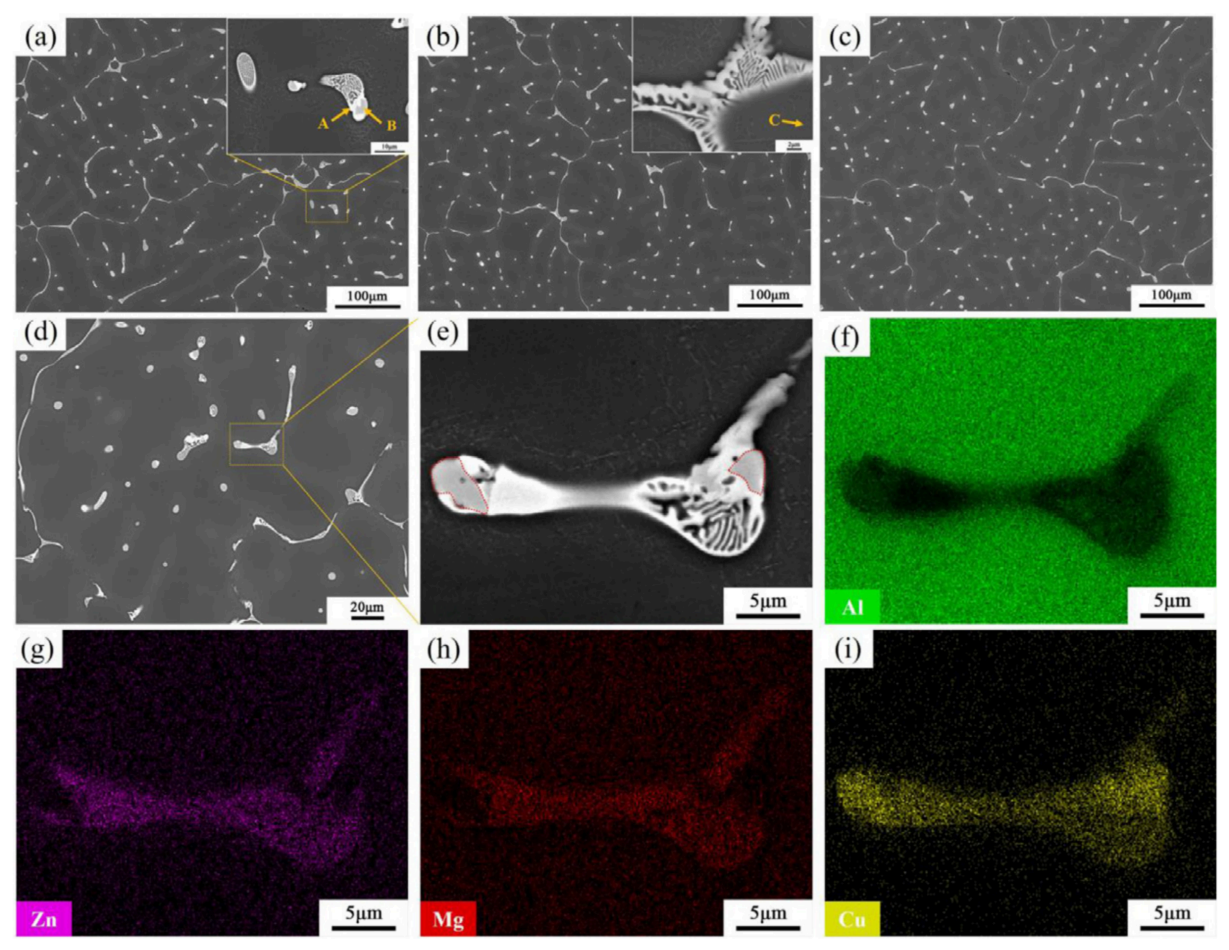
As-cast microstructures and elemental segregation in Al-Zn-Mg-Cu alloys with different Zn/Mg ratios (**a**) A1 alloy; (**b**) A2 alloy; (**c**) A3 alloy; (**d**) selected SEM region of the A3 alloy; (**e**–**i**) elemental segregation of the as-cast A3 alloy microstructures [109].

The content of Zn and the ratio of Zn to Mg determine the type and volume fraction of the second phase in the as-cast state, as well as the dissolution and transformation during the homogenization process. Increasing the alloying degree is beneficial for aging strengthening, but it will expand the solidification range and the degree of segregation [108,109,111]. Yin et al. [109] compared the as-cast microstructures and element segregation of Al-Zn-Mg-Cu alloys with different Zn/Mg ratios. The results showed that the Zn/Mg ratio significantly changed the inter-dendritic second phase and the element segregation characteristics, as shown in Figure 11. Squeeze casting and other pressure solidification methods can improve the feeding capability, reduce pores, and refine the microstructure [110], but they cannot eliminate the chemical segregation and continuous second phase at the grain boundaries caused by high alloying. Therefore, component design, pressure solidification, and subsequent heat treatment must be considered as an integrated scheme.

The most distinctive feature of Al-Zn-Mg-Cu is its high potential for aging strengthening, which is expected to create a new material space between Al-Si’s complex forming and 7xxx’s ultra-high strength. Its engineering bottlenecks are also the most concentrated, including hot cracking, segregation, corrosion, the solution treatment window, and the cost. Currently, it is more suitable for high-value-added squeeze casting or pressure casting.

### 4.5. Relationship Between Casting Process and Microstructure Properties

The casting process directly reconfigures the microstructure and defects of the same alloy by altering the filling speed, pressure, cooling rate, and feeding conditions. Gravity sand casting has a slower cooling rate and is suitable for large-scale and low-cost components, but the dendrites are coarser and more susceptible to feeding. Metal molds and low-pressure casting increase the cooling rate and make the filling process more stable, enabling a better density for components such as hubs. High-pressure die casting achieves the high-rate production of thin-walled components at a high speed, high pressure, and high cooling rate, but the risk of gas entrainment necessitates vacuum system control [89,91,112]. Squeeze casting solidifies under pressure, has strong feeding capabilities, and is suitable for components that require a high density and improved mechanical properties [110,113].

The essence of process selection lies in the regulation of three basic variables: the cooling rate, pressure, and flow state. Increasing the cooling rate usually helps to refine grains, form eutectics and intermetallic compounds, and reduce the microscopic segregation scale. Increasing the pressure is beneficial for feeding during solidification and reducing pores. A high-speed flow may increase oxide film entrainment and gas entrapment. Therefore, a high pressure does not necessarily equate to high performance. Only when the vacuum, pouring system, and melt quality are simultaneously optimized can the microstructural advantages of high cooling rates be transformed into stable performance.

The casting process also affects the available window for subsequent heat treatment. If a conventional high-pressure die-casting system contains excessive entrapped gas, it is prone to blistering and dimensional distortion during high-temperature solution treatment; therefore, conventional long-duration T6 treatment is restricted. Vacuum casting, short-term solution treatment, and low-temperature aging can alleviate this problem [84,89]. Squeeze casting and low-pressure casting are more suitable for full solution treatment and aging due to their lower porosity. The material grade and forming process cannot be chosen independently. The same nominal composition may exhibit completely different heat treatability and fatigue reliability under different processes. Table 3 presents a comparison of the microstructural and engineering characteristics of representative aluminum alloy casting processes.

### 4.6. Comprehensive Comparison of Different Casting Aluminum Alloy Systems

The advantages of different casting systems are highly complementary. Al-Si has the widest casting window and the strongest adaptability to complex thin-walled structures, making it the preferred choice for most industrial complex castings. Al-Cu stands out for its high strength and medium–high temperature potential, but segregation, thermal cracking, and corrosion resistance are the main costs. Al-Mg is outstanding in terms of seawater corrosion resistance, plasticity, and surface quality, but it faces limitations in melt oxidation, fluidity, and high-temperature strength. Al-Zn-Mg-Cu has one of the highest age hardening potentials, but high alloying makes the casting and heat treatment windows narrower. Table 4 summarizes the material positioning of the four systems.

As discussed in Section 4.2.3, the fatigue reliability of cast aluminum alloys is strongly influenced by the defect state and the distribution of critical defects [90,93]. On this basis, the strength–ductility trade-offs of different cast alloys can be further illustrated quantitatively within specific alloy and processing windows. For example, a high-strength Al-Si-Cu-Mg alloy processed by high-pressure die casting (HPDC) achieved yield strength of 321 MPa, ultimate tensile strength of 425 MPa, and elongation of 11.3% after solution treatment and aging [91]. In another Al-Si-Mn-Mg HPDC alloy, the as-cast ultimate tensile strength and elongation were 242–282 MPa and 2.6–4.7%, respectively, whereas, after T7 treatment, they were 195–207 MPa and 4.6–12.1% [92]. These results demonstrate that heat treatment may improve ductility at the expense of tensile strength and that quantitative comparisons should therefore be interpreted together with the alloy composition, casting route, heat treatment conditions, and defect state rather than on the basis of the nominal strength alone.

Overall, the technological evolution of cast aluminum alloys exhibits distinct differentiation characteristics. For Al-Si, the key lies in further reducing extreme defects and improving the adaptability of recycled raw materials. For Al-Cu, it is necessary to enhance the thermal stability and reduce segregation. For Al-Mg, it is necessary to address melt quality and high-temperature performance. For Al-Zn-Mg-Cu, a more reliable high-alloying casting window needs to be established. The quality of material design must be evaluated based on the specific processes, component wall thickness, and service conditions, rather than merely ranking them by the peak tensile strength of standard test bars.

## 5. Common Issues and Development Trends of Traditional Aluminum Alloys

The grades and manufacturing systems of traditional aluminum alloys have become relatively mature. The current research focus is more on multi-performance coordination, adaptability to recycled raw materials, design efficiency, and engineering reliability. These issues span different alloy series and directly affect the further upgrading of mature grades.

### 5.1. Multi-Performance Synergy and Precise Microstructural Control

The space for increasing strength through the addition of main alloying elements in mature aluminum alloys is limited. For 2xxx and 7xxx alloys, the enhancement of precipitation strengthening is constrained by grain boundary precipitation, quenching sensitivity, and corrosion. For 5xxx alloys, increasing the Mg content will increase the sensitization tendency. In Al-Si-Cu alloys, an increase in Cu content will exacerbate segregation and incipient melting risks. Therefore, performance evaluation should extend beyond average microstructural parameters to include local characteristics such as the grain boundary precipitation continuity, PFZ width, recrystallization fraction, residual stress, and extreme defects. The value of APT, TEM, EBSD, and XCT should also be better reflected in the quantitative correlations between these microstructural parameters and fatigue, corrosion, and fracture behaviors.

### 5.2. Design of Residual Element Limits for Recycled Aluminum

Recycled raw materials can increase the fluctuations of residual elements such as Fe, Si, Cu, and Zn, and the impurity limits of traditional grades are mostly based on higher-purity raw materials [9,10]. Under recycling-based manufacturing conditions, relying solely on deep purification will weaken the cost and energy-saving advantages of recycled aluminum. A more practical approach is to determine the acceptable range of residual elements and evaluate their hazards in combination with the type, size, and morphology of the second phase. The morphology of Fe-rich phases in Al-Si alloys can be controlled through Mn addition and solidification conditions, and, in some Al-Mg-Mn die-casting systems, the controlled Fe may also have a certain strengthening effect [106,107].

For wrought alloys, it is also necessary to consider the influence of coarse residual particles on rolling, fatigue, and corrosion. The 6xxx series has a wide composition range and is an important target for automotive closed-loop recycling. Some studies have begun to conduct classification and recycling design by simultaneously using composition, process, and multiple performance constraints [114,115]. Raw material sorting, composition compensation, and solidification and heat treatment control should form a continuous quality control chain, enabling the simultaneous achievement of a higher recycling rate and stable microstructure.

### 5.3. Computational and Data-Driven Alloy Design

CALPHAD can be used to screen the phase stability regions, solidification paths, precipitation driving forces, and non-equilibrium solidification trends of multi-component aluminum alloys. It can also be combined with Scheil-type solidification calculations, diffusion and precipitation kinetics models, and phase-field simulations to narrow the experimental and processing space [116,117]. Machine learning is suitable for handling high-dimensional compositions and process spaces. Relevant studies have covered the screening of high-strength aluminum alloys, transfer learning with small datasets, and the prediction of commercial wrought aluminum alloys [118,119,120], as well as feature engineering, multi-objective reverse design, and classification [121,122,123].

At the thermodynamic level, CALPHAD represents each phase using Gibbs energy models and determines equilibrium states by minimizing the total Gibbs energy of a multi-component system. Its simplified form is G = Σ_i_x_i_G_i_^0^ + RTΣ_i_x_i_ln x_i_ + G^ex^, where G_i_^0^ denotes the reference state contribution and G^ex^ accounts for non-ideal interactions [116,117]. This thermodynamic description provides the basis for evaluating phase stability and transformation driving forces over multi-component composition spaces.

However, the reliability of CALPHAD calculations is limited by the evaluated database and its associated physical models. When the composition, temperature, metastable phase, interfacial energy, or mobility data are outside the calibrated range, the reliability of the prediction results will decrease. Moreover, relying merely on the equilibrium phase composition prevents the reproduction of phenomena such as transient precipitation, segregation, quenching rate effects, or defect formation. Therefore, the sensitivity to the selection of the database and the uncertainty of the model parameters should be reported as much as possible. Before being applied to industrial products, the calculated phase and processing window should be compared with the actual measured microstructure and performance [116,117].

In casting alloys, data-driven methods have been used to predict macroscopic shrinkage and support thermodynamic-assisted alloy design [124,125]. Additionally, other related studies focus on age hardening and the interpretable modeling of heat-resistant alloys [126,127]. These applications demonstrate the wide applicability of data-driven screening. However, the proposed compositions and processes still need to be verified through experiments, and descriptors with physical significance should be retained rather than relying solely on statistical accuracy [118,119,123].

There is another related limitation in machine learning prediction, namely the transferability of the fitted function relationship Ŷ = f(x; θ), which depends on the training domain, represented by the components, heat treatment, product shape, thickness, and test conditions. Small or heterogeneous literature datasets may contain reporting biases, related records under the same alloy or tempering state, and missing process variables. Therefore, randomly dividing the training and test sets may lead to the overestimation of the model’s generalization ability. In addition to the conventional prediction error, external validation, single-alloy exclusion tests, uncertainty estimation, and cross-domain checks are also crucial when the model is used for reverse design or screening [118,119,120,121,122,123]. Explainable machine learning methods can identify influential descriptors, but the importance of features should not be interpreted as a causal metallurgical mechanism without independent verification [127].

A more robust approach is to combine these two strategies. CALPHAD or physically derived quantities can be used as constrained descriptors for data-driven models, while machine learning can prioritize experiments within the physically feasible composition and process space [125,126,127]. The final evaluation criteria should not only be based on the highest cross-validation score but also focus on whether the candidate alloys and processes still maintain accuracy in independent experiments and whether the predicted trends still have physical interpretability.

### 5.4. Scale-Up, Reliability, and Standardization from Laboratory Samples to Engineering Components

Whether laboratory results can be translated into engineering performance mainly depends on the size effect and manufacturing fluctuations. There are significant differences in cross-sectional thermal histories for thick plates, forgings, and large-section extruded components, and the quenching sensitivity of high-strength 7xxx is a typical example [48]. For large-scale products, the microstructure, strength, corrosion resistance, and residual stress at different thickness positions should be compared, and a single sampling location should not be used to represent the overall state.

The wall thickness and geometry of the casting will alter the filling, feeding, and solidification paths, and the porosity, segregation, and oxide films will also be spatially non-uniformly distributed. XCT can be used to identify critical pores and their locations [93], and, for high-pressure die casting, the vacuum level, melt quality, and heat treatment window should also be evaluated [112]. Engineering judgment should focus more on extreme defects, batch fluctuations, and long-term fatigue and corrosion stability, so that the optimization results for the microstructure can be repeatedly obtained under real components and production conditions.

To avoid overgeneralization, evidence should be classified based on the level of verification. Small-scale experiments are suitable for identifying mechanisms, and pilot-scale tests can be used to reveal thermal gradients and defect distributions, while full-component or industrial tests are needed to verify their repeatability under real conditions of quenching, filling, heat treatment, and raw material recovery changes. Similarly, CALPHAD or machine learning-assisted design should also follow the same verification levels. Therefore, statements regarding scale-up applications should clearly specify the sample or component size, processing boundary conditions, sampling location, and whether the verification is internal, external, or component-level.

## 6. Conclusions

The microstructure control of traditional aluminum alloys is governed by the manufacturing process. For wrought alloys, the grain size, texture, dislocations, and precipitation states are adjusted through plastic processing and heat treatment, while cast alloys are more influenced by the initial solidification microstructure and defects such as intermetallic compounds, pores, and oxide films. There is no unified performance ranking for different series. For 2xxx and 7xxx, the focus is on high-strength load-bearing applications; for 5xxx, corrosion resistance and weldability are emphasized; for 6xxx, overall manufacturability is emphasized; and 3xxx, 4xxx, and representative 8xxx alloys serve specific scenarios such as forming, joining, and foil materials. Cast alloy systems involve trade-offs between casting properties, strength, corrosion resistance, and thermal stability.

The focus of improving mature aluminum alloys has shifted from the peak strength to microstructure stability and service reliability. Nanoscale precipitation, grain boundary states, recrystallization, coarse secondary phases, three-dimensional defects, and residual stress should be evaluated in combination with specific components, and the analysis of fatigue and corrosion issues should particularly focus on local extreme values and their statistical distribution.

High proportions of recycled raw materials increase the difficulty of composition and second phase control, and designs aimed at impurity tolerance have more engineering significance. CALPHAD, physical models, and machine learning can improve the screening efficiency, but they still require experimental and component-level verification. For mature aluminum alloys, it is necessary to pay more attention to performance stability under higher recycled content and complex service conditions.

## Figures and Tables

**Table 1:** Major alloying elements, representative phases, and microstructural roles in conventional aluminum alloys.

Element	Typical Systems	Representative Phases/Constituents	Main Roles	Potential Drawbacks
Cu	2xxx; cast Al-Cu and Al-Si-Cu alloys	θ″, θ′, θ-Al_2_Cu, some S-type phases	High precipitation strengthening potential, improves intermediate-temperature strength	Segregation, reduced corrosion resistance, narrower solution treatment window
Mg	5xxx, 6xxx, 7xxx, cast Al-Mg alloys	Solid solution strengthening, β″-type phases, η-type phases, β-Al_3_Mg_2_ during sensitization	Increases strength, work hardening capacity, and age hardening response	Oxidation of high-Mg melts, grain boundary sensitization in 5xxx alloys
Si	4xxx, 6xxx, cast Al-Si alloys	Eutectic Si, Mg-Si precipitates	Improves fluidity and joining performance, contributes to strengthening in 6xxx alloys	Coarse Si reduces ductility, affects the types of Fe-rich phases
Zn	7xxx, cast Al-Zn-Mg-Cu alloys	GP zones, η′, η-MgZn_2_	Enables very high age-hardening response	Segregation, stress corrosion susceptibility, quench sensitivity
Mn/Cr	3xxx, recycled cast alloys	Al(Mn,Fe)Si dispersoids and related phases	Dispersion strengthening, suppresses recrystallization, modifies Fe-rich phases	Excessive additions may promote coarse intermetallic compounds
Zr/Sc/Ti	High-strength wrought and high-performance cast alloys	Al_3_Zr, Al_3_Sc, and related dispersoids	Grain refinement and improved microstructural thermal stability	Cost, formation of coarse primary phases, processing compatibility
Fe	Various recycled aluminum alloys	Fe-rich intermetallic compounds	May contribute to dispersion or load-bearing effects when morphology is controlled	Plate- or needle-like brittle phases, fatigue, and surface defects

**Table 2:** Property positioning and engineering selection of major wrought aluminum alloy systems.

Alloy System	Strengthening Characteristics	Relative Strength	Key Advantages	Main Limitations	Typical Applications
2xxx	θ- and S-type precipitation, Mn/Zr dispersoids	High	Good fatigue resistance, damage tolerance, and intermediate-temperature performance	Corrosion resistance, coarse particles, residual stress in thick plates	Aircraft skins, structural plates, welded aerospace components
3xxx	Mn solid solution and Al(Mn,Fe)Si dispersoids	Low–medium	Formability, corrosion resistance, low cost, and heat exchanger suitability	Limited strength, sensitivity to Fe/Si variation	Heat exchangers, packaging, building products
4xxx	Si phases, limited precipitation strengthening	Low–medium	Low-melting joining capability and wear resistance	Limited load-bearing capacity	Filler wires, brazing materials, wear-resistant components
5xxx	Mg solid solution and work hardening	Medium	Seawater corrosion resistance, weldability, and low-temperature toughness	β-phase sensitization, limited high-temperature strength	Ships, storage tanks, welded transport structures
6xxx	β″ precipitation strengthening	Medium–high	Balanced formability, corrosion resistance, surface quality, and cost	Natural aging, quench sensitivity, weld softening	Automotive sheet, extrusions, rail transit structures
7xxx	GP zones and η′ precipitation, Zr dispersoids	Very high	Highest strength potential among conventional wrought aluminum alloys	SCC susceptibility, quench sensitivity, through-thickness uniformity	Primary load-bearing aerospace thick plates and forgings
8xxx Al-Fe-Si	Fe-rich phases and work hardening	Low–medium	Ultra-thin rolling, low cost, and packaging functionality	Pinholes, surface quality, sensitivity to second-phase particles	Food/pharmaceutical foils, thermal management foils

**Table 3:** Microstructural and engineering characteristics of representative aluminum alloy casting processes.

Casting Process	Filling and Solidification Characteristics	Microstructural Features	Defect and Heat Treatment Characteristics	Typical Applications
Gravity sand or permanent-mold casting	Low pressure, low-to-moderate cooling rate	Relatively coarse dendrites and eutectic constituents	Sensitive to feeding-related shrinkage and porosity, full T6 treatment is generally applicable	Large housings, conventional engine components, general-purpose castings
Low-pressure casting	Stable bottom-up filling with sustained feeding	Comparatively uniform microstructure	Low gas entrainment, good heat treatment compatibility	Wheels and complex load-bearing castings
High-pressure die casting (HPDC)	High-speed, high-pressure filling, high cooling rate	Fine grains and eutectic constituents, strong thin-wall capabilities	Gas entrainment requires vacuum control, conventional high-temperature solution treatment is restricted	Automotive integrated structures and thin-wall housings
Squeeze casting	Solidification and feeding under applied pressure	Dense and relatively fine microstructure	Low porosity, suitable for solution treatment and aging	High-strength, thick-wall load-bearing components

**Table 4:** Strengthening characteristics, advantages, and engineering limitations of major cast aluminum alloy systems.

Alloy System	Major Microstructural Constituents and Strengthening Phases	Primary Advantage	Main Limitations	Typical Applications
Al-Si	α-Al, eutectic Si, Mg-Si, θ-type and Q-type phases	Best overall castability and complex shape capabilities	Eutectic Si, Fe-rich phases, porosity, and fatigue scatter	Automotive housings, wheels, powertrain components, integrated structures
Al-Cu	θ-type Al_2_Cu; selected S-type phases	High strength and intermediate-to-high-temperature potential	Segregation, hot tearing, narrow solution treatment window, and corrosion susceptibility	Aerospace and powertrain heat-resistant complex castings
Al-Mg	Mg solid solution; Sc/Zr/Er-containing dispersoids	Seawater corrosion resistance, toughness, and surface quality	Oxide inclusions, limited fluidity, and limited high-temperature strength	Marine, instrument, and corrosion-resistant housings
Al-Zn-Mg-Cu	η′/η and Mg-Zn-Cu-related phases	High strength potential and age-hardenability	Segregation, hot tearing, corrosion susceptibility, and narrow heat treatment window	High-strength pressure- and squeeze-cast components

## Data Availability

No new data were created or analyzed in this study. Data sharing is not applicable to this article.
